# Single-pixel Fresnel incoherent correlation holography compressed imaging using a Trumpet network

**DOI:** 10.1038/s41598-024-64673-6

**Published:** 2024-06-14

**Authors:** Jiaosheng Li, Yifei Chen, Tianyun Liu, Bo Wu, Qinnan Zhang

**Affiliations:** 1 School of Photoelectric Engineering, Guangdong Polytechnic Normal University, Guangzhou, 510665 China; 2 School of Electronics and Information, Guangdong Polytechnic Normal University, Guangzhou, 510665 China

**Keywords:** Imaging and sensing, Interference microscopy

## Abstract

Fresnel incoherent correlation holography (FINCH) can achieve high-precision and non-scanning 3D imaging. However, as a holographic imaging technology, the huge bandwidth requirements and the amount of holographic data transmitted have always been one of the important factors limiting its application. In addition, the hardware cost of pixel array-based CCD or CMOS imaging is very high under high resolution or specific wavelength conditions. Accordingly, a single-pixel Fresnel incoherent correlation holography (SP-FINCH) compressed imaging method is proposed, which replaces pixel array detector with single-pixel detector and designs a Trumpet network to achieve low-cost and high-resolution imaging. Firstly, a modified FINCH imaging system is constructed and data acquisition is carried out using a single-pixel detector. Secondly, a Trumpet network is constructed to directly map the relationship between one-dimensional sampled data and two-dimensional image in an end-to-end manner. Moreover, by comparing the reconstructed images using neural network with that using commonly used single-pixel reconstruction methods, the results indicate that the proposed SP-FINCH compressed imaging method can significantly improve the quality of image reconstruction at lower sampling rate and achieve imaging without phase-shifting operation. The proposed method has been shown to be feasible and advantageous through numerical simulations and optical experiment results.

## Introduction

Quantitative phase imaging (QPI) is a method that utilizes the principle of light interference to quantitatively measure the phase changes caused by the optical path changes of the sample, and thereby obtain 3D information of the sample^1,2^. Quantitative phase measurement techniques using coherent illumination often produce speckle and parasitic fringes, which will significantly reduce image quality and limit their practical applications. The development of digital holography based on self-interference theory breaks the reliance of traditional digital holography on laser illumination. Fresnel incoherent correlation holography (FINCH)^3^ is a technology that utilizes self-interference theory to achieve non-scanning 3D imaging. By loading spatial light modulators (SLM) with different lens phases, it cleverly constructs an on-axis self-correlation optical path to record holograms containing the sample information in 3D space, which has been widely studied and applied in other microscopic imaging fields due to its non-scanning characteristics and super-resolution advantages^4,5^. The existing FINCH imaging technology has made significant progress, such as those based on checkerboard lens^6^, SLM^7,8^, and single-exposure polarization-encoded phase-shifting digital holography^9,10^, which can effectively eliminate reconstruction noise and achieve fast imaging speed. Additionally, various schemes have been proposed to address issues such as phase-shift error correction and edge contrast, etc.^4,11,12^. Due to its simple system structure, ease to phase shift, and low reconstruction noise, incoherent digital holography based on SLM has achieved fluorescent 3D imaging and increasing attention^13^. However, to date, the large bandwidth requirement and the amount of transmitted hologram data have been important factors limiting the application of digital holography. Moreover, the conventional holographic imaging technique, which relies on pixel array-based CCD or CMOS, incurs substantial hardware expenses especially under high-resolution or specialized wavelength requirements.

Single-pixel imaging (SPI) is an imaging technology that modulates the light field of the target object with different illumination patterns successively, records the one-dimensional (1D) light field signal with a single-pixel sensor without spatial resolution, and then reconstructs the sample based on the correlation between the illumination pattern and the single-pixel signal^14,15^. Compared to traditional imaging methods, SPI uses relatively inexpensive single-pixel detector instead of pixel-array detector, particularly advantageous in non-visible light bands due to cost advantages. It can also complete tasks that are difficult for traditional cameras to accomplish, such as non-line-of-sight imaging and weak light imaging. Additionally, the introduction of compressed sensing can greatly reduce the measurement times in SPI, allowing for clear images to be reconstructed from under-sampled data, thereby increasing the speed of SPI system^16,17^. It also provides an effective way to solve the problem of large amounts of data in digital holography methods. However, it has been found from previous research that the data amount required for holographic reconstruction is mutually restrictive with the quality of image reconstruction due to limitations in measurement matrices or reconstruction algorithms and experimental conditions, posing challenges in practical applications. Recently, deep learning (DL) has shown strong feature learning and expression capabilities, enabling it to achieve better image reconstruction quality with lower computational complexity than traditional methods^18–20^. Among them, convolutional networks have demonstrated their powerful capabilities in optical holographic reconstruction^21,22^. Currently, DL methods have been applied to the field of SPI to address limitations in imaging quality, system noise, and reconstruction speed for SPI technology^23,24^.

Accordingly, we develop a single-pixel Fresnel incoherent correlation holography (SP-FINCH) compressed imaging method by combining DL with SPI technology. This method utilizes a single-pixel detector instead of a pixel-array detector and designs a Trumpet network to achieve low-cost and high-resolution imaging. It further addresses the limitations of the large bandwidth requirement and the transmission of hologram data for the incoherent digital holography technology. Both simulation and experimental analysis have demonstrated that this approach is capable of effectively minimizing the amount of hologram data while maintaining a high level of reconstruction quality, and its benefits become even more evident with decreased sampling rates. The proposed SP-FINCH compressed imaging method not only breaks through the limitations of array sensors, but also possesses the capacity to be expanded to other imaging bands, which can expand the scope of FINCH application research in the area of 3D imaging.

## Method implementation and network analysis

**Figure 1:**
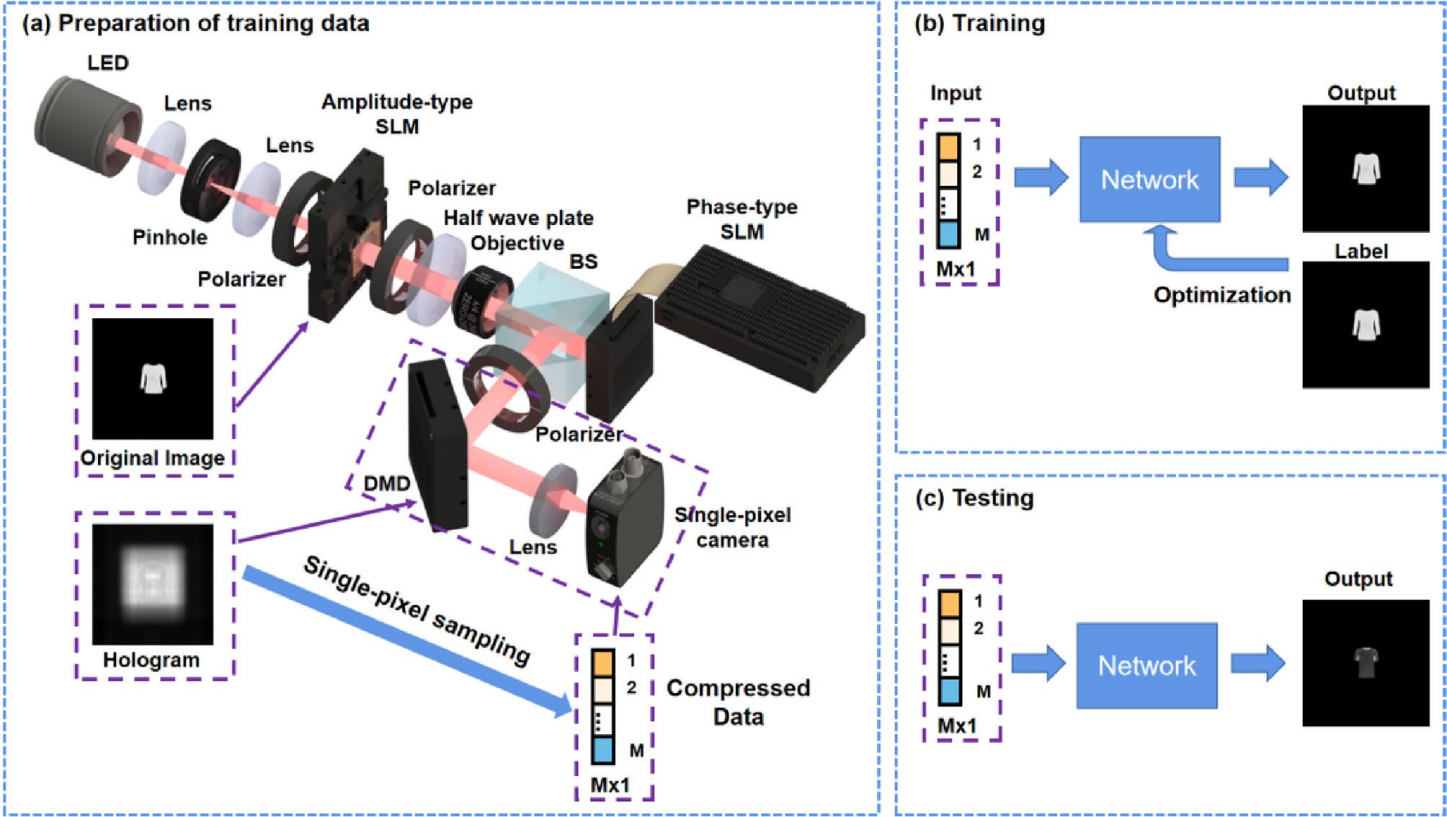
The flow chart of the SP-FINCH compressed imaging method. (**a**) Preparation of training data; (**b**) Training and (**c**) Testing process, in which the SLM and DMD refer to spatial light modulator and digital micromirror device.

The entire SP-FINCH imaging process involves the preparation of training data, training, and prediction processes, as detailed in Fig. 1. Firstly, incoherent imaging is achieved by using a modified FINCH imaging system, in which a amplitude-type SLM (Holoeye, LC 2012) and a phase-type SLM (Holoeye, PLUTO) are used for loading the original image and lens phase, respectively. Then, the compressed 1D data is generated by projecting the measurement matrix onto the digital micromirror device (DMD) with the experimental hologram. Later on, a Trumpet network is designed for training together with the corresponding label image. Lastly, the information is rebuilt with high quality using only a set of compressed 1D data. The detailed execution process and network structure analysis will be addressed in the "Method implementation" and "Network analysis".

### Method implementation

In this section, we combine the modified FINCH imaging system to modulate the original image with a loaded measurement matrix on the DMD. At this time, the form of the hologram projected onto the DMD plane can be represented as

1 $$I(x_{D} ,y_{D} ) = C\iiint {o(x_{o} ,y_{o} ,z)\left\{ {1 + \frac{1}{2}\exp [i\varphi (x_{D} - x_{o} ,y_{D} - y_{o} ,z)] + \frac{1}{2}\exp [ - i\varphi (x_{D} - x_{o} ,y_{D} - y_{o} ,z)]} \right\}dx_{o} dy_{o} dz},$$

where $O(x_{o} ,y_{o} ,z_{o} )$ represents the intensity of the input object function of the system, $C$ is a constant containing information about the intensity of the object points, *i* is the imaginary unit, and $\varphi (x_{D} - x_{o} ,y_{D} - y_{o} ,z) = {{\pi \left[ {(x_{D} - x_{o} )^{2} + (y_{D} - y_{o} )^{2} } \right]} \mathord{\left/ {\vphantom {{\pi \left[ {(x_{D} - x_{o} )^{2} + (y_{D} - y_{o} )^{2} } \right]} {(z\lambda )}}} \right. \kern-0pt} {(z\lambda )}}$ indicates a quadratic phase factor that encodes information about the depth and lateral position of the object points. *x*_o_, *y*_o_, *z*_o_ represent the coordinates of the object, while $x_{D}$ and $y_{D}$ represent the coordinates on the DMD plane. While forming a hologram on the DMD, linear measurement of the holographic data and the measurement matrix loaded on the DMD is completed through the reflection of the DMD, obtaining a measurement value:

2 $$y_{m} = \langle \varphi_{m} ,I\rangle ,m \in \left\{ {1,2, \ldots M} \right\},$$

in which $y_{m}$ is the *m*-th measurement value, and $\varphi_{m}$ is the *m*-th pseudo-random measurement matrix generated by the DMD. Then by repeating *M* times and utilizing the weighted effect of the single-pixel detector, the corresponding measurement values can be obtained by.

3 $$Y = \left[ {y_{1} \, y_{2} \, ... \, y_{m \, } ... \, y_{M} } \right] = \langle \Psi ,I\rangle ,$$

where, $\left\langle \cdot \right\rangle$ indicates the inner product operation and $\Psi \in R^{M \times N}$ denotes the measurement matrix. After acquiring the corresponding 1D data, the established network can be utilized for training purposes, aiming to derive the mapping correlation between the 1D data and the two-dimensional (2D) image, this process is represented as:

4 $$O^{\prime} = \Theta_{{{\mathbf{Trumpet}}}} (Y).$$

In this process, the reconstructed image during training is denoted as $O^{\prime}$, and a nonlinear mapping function is denoted as $\Theta_{{{\mathbf{Trumpet}}}} ()$, which is based on the designed network model that transforms 1D sampling data into the 2D image space through the Trumpet network that is specifically designed for this purpose.

### Network analysis

**Figure 2:**
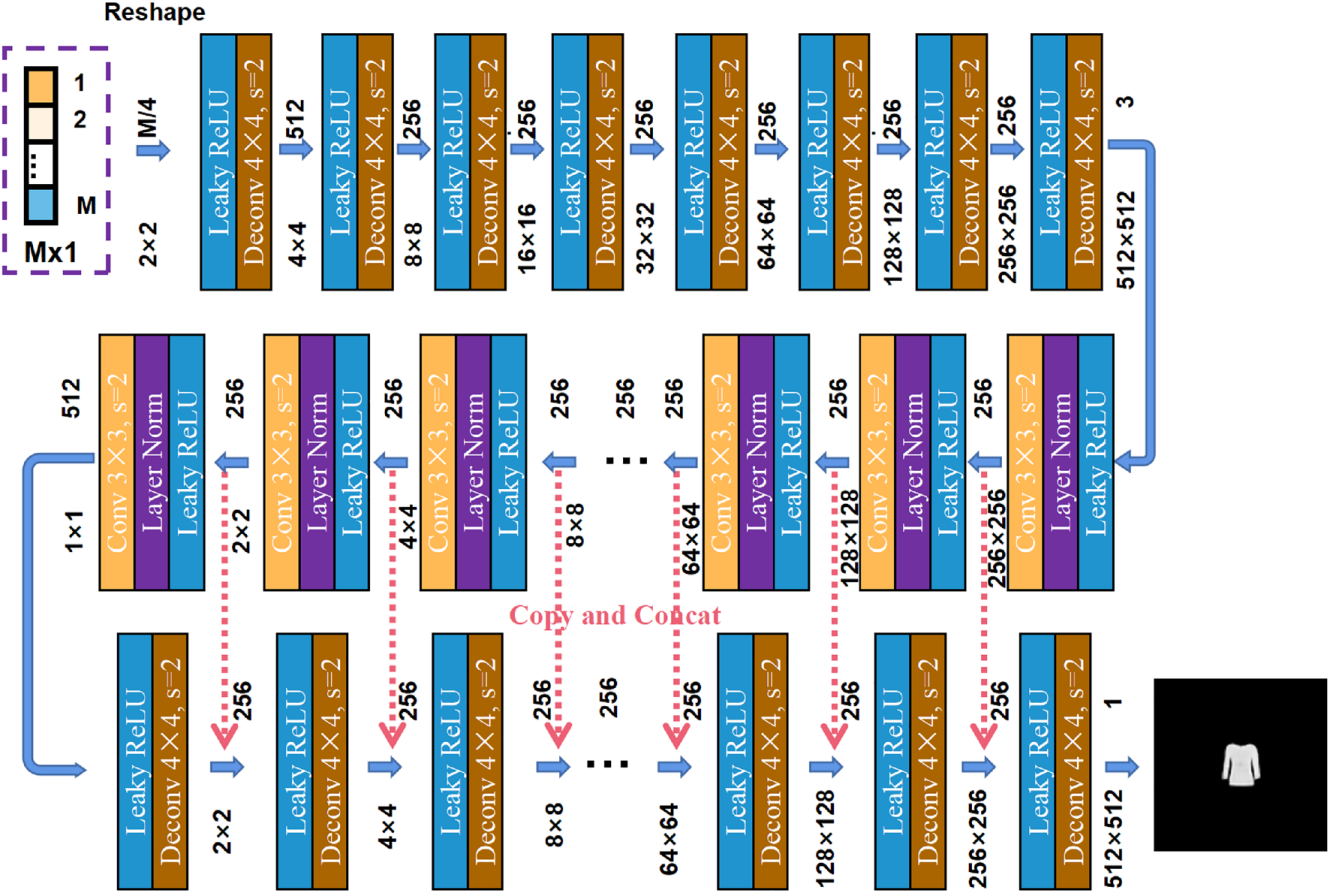
The designed “Trumpet” neural network for mapping between 1D data and 2D image.

Based on the proposed approach, we developed a “Trumpet” neural network for mapping between 1D data and 2D image, as shown in Fig. 2. Firstly, the network is designed to map the 1D signal of size 1 × 1 × M to the 2D signal of size 2 × 2 × N, where *N* = *M*/4. When *M* is not a multiple of 4, a zero-padding operation needs to be performed at the output end of the signal. This operation can greatly reduce the number of network parameters. Subsequently, multi-layer deconvolution layers are used for the up-sample of the collected signal. The first deconvolution layer converts 1D signal into 512-layer feature signal with a size of 4 × 4, using a kernel size of 4 and a step size of 2. Then, the feature signal is upconverted by using the 8 deconvolution layers. Each deconvolution layer consists of a deconvolution operation and leaky ReLU layer. The input 1D signal is initially converted into feature maps with a size of 3 × 512 × 512. Subsequently, the Unet network is used to further process the upsampled feature maps to enable more accurate reconstruction results. The latter part of the network structure consists of 8 convolutional layers and 8 deconvolutional layers, with the parameters of the convolutional and deconvolutional layers shown in the Fig. 2, in which the convolutional layer utilizes a 3 × 3 convolution kernel with a step size of 2. Similarly, every convolution layer consists of a convolution operation, a Batch Normalization (BN) layer and leaky ReLU layer. In deconvolution operation, the kernel size is 4 × 4 and the step size is 2. Each deconvolution layer consists of a deconvolution operation and leaky ReLU layer. The feature maps from each convolution layer in the Unet network are copied and contacted to the corresponding deconvolution layer. All programs are executed in a Python 3.9 environment using the PyTorch command, and the operation is accelerated by utilizing a NVIDIA GeForce GTX 4090Ti GPU. The training step is 200.

## Simulation results and performance analysis

**Figure 3:**
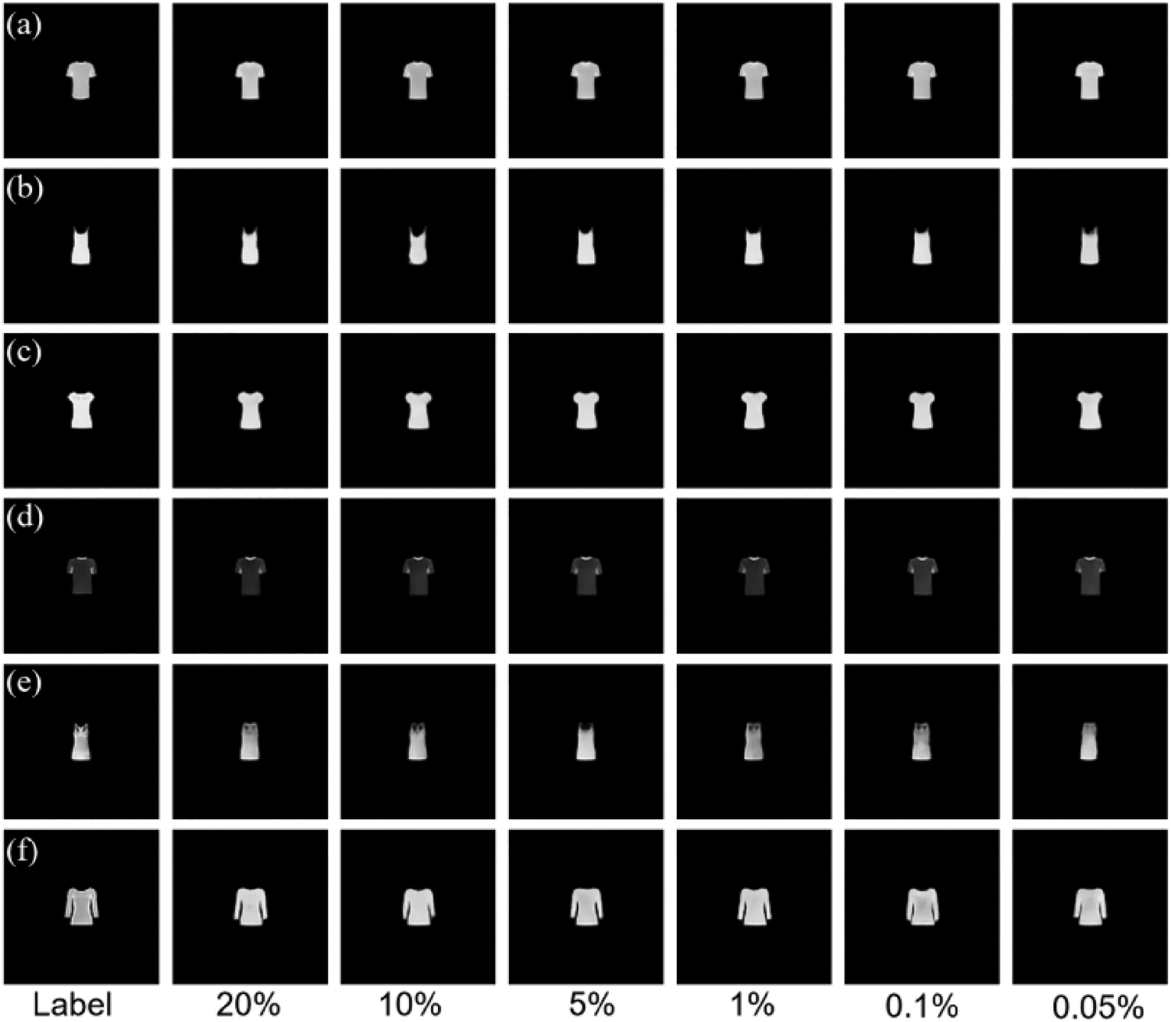
Simulation reconstruction results of different sets of image at different sampling rates.

**Figure 4:**
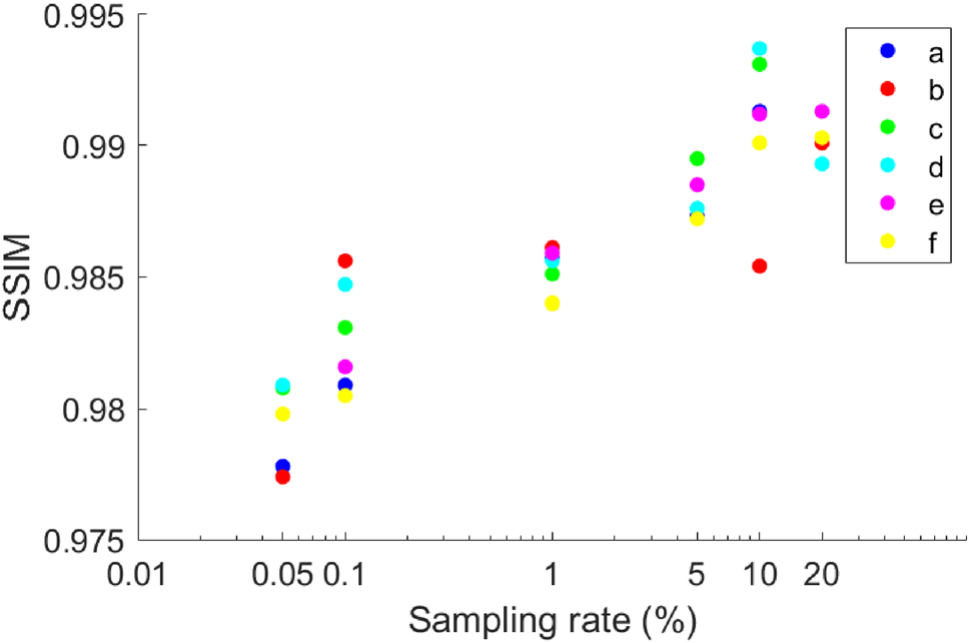
Distribution of SSIM values for different groups of reconstruction image at different sampling rates.

We first tested the proposed SP-FINCH method using the Fashion-MNIST dataset, using 6000 pairs of data for training, with 80% serving as the training set, 10% serving as the testing set, and the remaining 10% serving as the validation set. The inner product operation between the hologram and the measurement matrix is performed according to Eq. (2), ultimately gathering the one-dimensional data at different sampling rates, in which the measurement matrix is a random binary matrix. Figure 3 shows the partial reconstruction results of six different sets of original images at sampling rates of 20%, 10%, 5%, 1%, 0. 1%, and 0.05%. Upon comparison with the label image, the reconstruction results achieved at the aforementioned sampling rates are visually indistinguishable from the label image to the naked eye. The reconstruction results exhibit virtually no changes, even in very low sampling rate (i.e. 0.05% sampling rate). To quantitatively analyze this set of results, the structural similarity index (SSIM) values are calculated and their distributions are shown in Fig. 4. From the distribution results, it found that the SSIM values of different groups at different sampling rates are distributed between 0.976 and 0.994, and with the increase of data volume, the SSIM values have a tendency to increase, but the overall range of variation is small, indicating that the proposed method can reconstruct the collected 1D data well. Especially when the sampling rate is extremely low, its SSIM value remains above 0.975, still retaining high accuracy, demonstrating the definite feasibility and superiority of the proposed SP-FINCH compressed imaging method.

## Experimental verification and performance on single-pixel reconstruction methods

**Figure 5:**
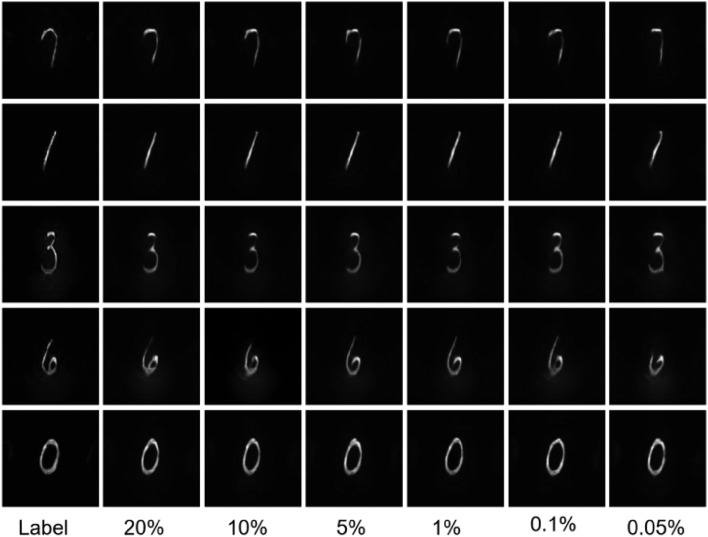
Experimental reconstruction results of different groups of numbers at different sampling rates.

Furthermore, we utilize experimental data collected from the SP-FINCH imaging system in Fig. 1a for training and prediction. The illumination is provided by an LED light source with a central wavelength of 625 nm. The beam is focused through a lens and then removed from stray light interference through an aperture diaphragm. Then, another lens is placed at the focal length to modulate the beam into collimated light. The amplitude-type SLM (Holoeye, LC 2012) is used to load the handwritten MNIST dataset, and the phase-type SLM (Holoeye, PLUTO) is used to implement encoding and phase-shifting. Similar to the simulation, the DMD (D4300) performs an inner product operation with the hologram after loading the measurement matrix, ultimately gathering the signal using a single-pixel detector. Then the images at sampling rates of 20%, 10%, 5%, 1%, 0.1%, and 0.05% are reconstructed, respectively. Where the label images are the results of CCD camera using traditional array imaging method. The corresponding experimental reconstruction results exhibited in Fig. 5 show that the reconstruction quality is high for all data at different sample rates. Only the fourth group of numbers, 6, is relatively poor when the sampling rate was 0.05%, while the rest retained high accuracy, which also further demonstrated the superiority of the proposed method experimentally.

**Figure 6:**
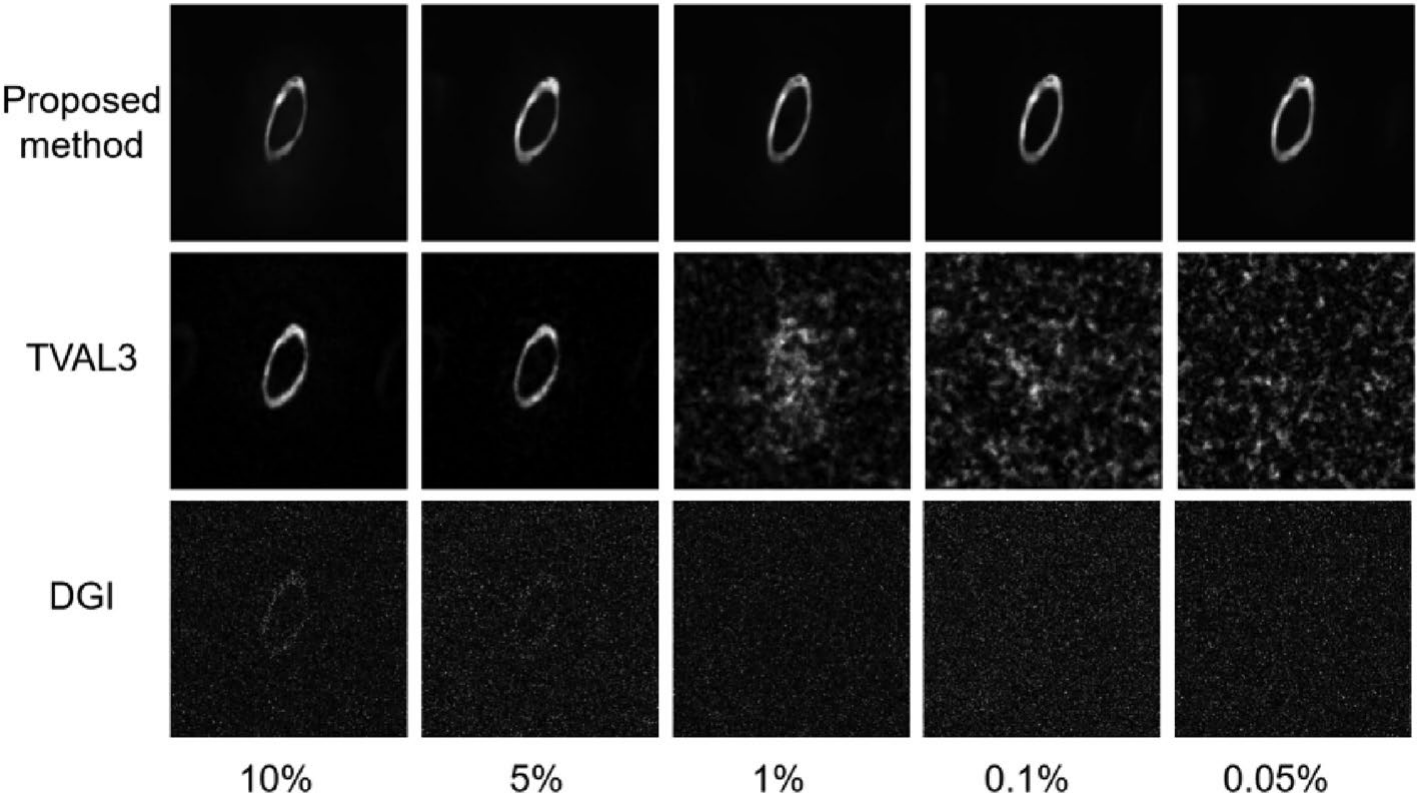
Reconstruction results of the number ‘0’ at different sampling rates using different reconstruction methods.

In addition, to compare with traditional single-pixel reconstruction methods. In this section, we utilized the fast and high-precision compression reconstruction method, known as the total variation minimization by augmented Lagrangian and alternating direction algorithm (TVAL3)^25^, together with the differential ghost imaging (DGI)^26^, to reconstruct a set of experimental data at different sampling rates, as displayed in Fig. 6. The reconstruction results presented in the first row are similar to the previous ones, which still yield good reconstruction performance at different sampling rates. The second row shows the reconstruction results obtained using TVAL3 algorithm. Evidently, the reconstruction accuracy of the TVAL3 algorithm can achieve good results when the sampling rate is above 5%, but when the sampling rate is below 1%, the reconstruction results are almost indistinguishable. In the third row, the reconstruction results using the DGI algorithm show that when the sampling rate is 10%, the signal-to-noise ratio of the algorithm is already very low, and at lower sampling rates, the results are almost indistinguishable. The above results further demonstrate that the proposed SP-FINCH imaging method combined Trumpet network has higher accuracy and more stable performance compared to traditional SPI reconstruction methods.

## Conclusion

In this paper, we presented a single-pixel Fresnel incoherent correlation holography (SP-FINCH) compressed imaging method using a Trumpet network with the aim of achieving low-cost and high-resolution imaging. The proposed method uses a single-pixel camera instead of a traditional pixel array-based camera to build a modified FINCH imaging system, and combines deep learning reconstruction method to directly reconstruct high-quality 2D original images from the collected 1D data. Moreover, compared to commonly used single-pixel reconstruction methods, the proposed method achieves better and more stable performance for image reconstruction at lower sampling rates. Simulation and experimental performance analysis demonstrate that the proposed method is expected to be expanded to a wider application range and further address the limitations of bandwidth and other factors on holographic data transmission and imaging field.

## Data Availability

The datasets generated during and/or analysed during the current study are available from the corresponding author on reasonable request.
